# Hygienic and Dietetic Treatment of Gonorrhea

**Published:** 1905-11

**Authors:** Henry G. Spooner

**Affiliations:** New York, 333 Second Avenue


### Hygienic and Dietetic Treatment of Gonorrhea.

By HENRY G. SPOONER, M. D., of New York.

GONORRHEA is a self-limited disease, that under the most favorable conditions of hygiene and diet, runs its course in five to eight weeks without any kind of treatment. To prove this fact, it is only necessary for the physician to put his patient to bed at the beginning of the florid stage of the disease; to feed him on a vegetable and fruit diet; to applv cold compresses to the sexual organs and to the perineum for several hours a day; to keep the bowels acting freely, and at the end of five to eight weeks, the gonorrhea will have run its course without complications.

I have treated eight cases in this way with the result stated. Experience has taught me that all injections and internal medication, are of little or no use, if the diet and hygiene of the patient are not carefully controlled. Unfortunately the majority of patients cannot remain in bed, as they must attend to their business, and have a natural desire to preserve the utmost secrecy about their condition.

The most important service the doctor can render his patients in any of the stages of clap, is to dominate their will so as to keep them physically and mentally as quiet as possible.

A good suspensory should be ordered, one with straps, to support the testicles and to keep the perineal part of the suspensory from constricting the urethra, thus avoiding congestion of this canal. This is intended to raise the penis and scrotum and to fix them upon the lower part of the abdomen, thereby preventing the shaking that tends to increase the inflammation. Any compression upon the perineum must be carefully avoided as this constricts the canal of the urethra and causes stasis of the secretion.

It can be readily understood that coitus during the course of the disease must be absolutely forbidden. The patient must be made to realise the infectious nature of the disease, even after the discharge has considerably lessened, since he does not see the reason for avoiding coitus as clearly as the doctor does; nor does he realise that the inflammation may augment the sexual impulse. Masturbation, gay female society, and lascivious pictures must be prohibited.

Aside froom the danger of infecting others, the patient must be warned to wash and sterilise his hands after touching his genitals, lest he infect his eyes.

The bed of the patient should not be too soft or warm, as these conditions favor erections. Gonorrhea produces great sexual excitement, manifested by erections and pollutions, which are extremely injurious. These should be combatted by a cool, hard bed, regulation of the bowels and the use of anaphrodisiacs. The internal administration of camphor and potassium bromide is also useful.

The glans and prepuce should be washed with soap and water and with mild astringents to remove the pus that constantly gathers. To prevent soiling the linen, gauze should be placed around the glans, so that when the prepuce is drawn forward, the gauze is held in place. Absorbent cotton should never be used for this purpose, as it does not permit of sufficient drainage. In private practice I have always used a D. A. B. D. gonorrhea apron.

In diet, everything is to be avoided that makes digestion slow, causes constipation, or that has an irritating effect on the urine, or sexual organs.

It is advisible to give each patient a printed or written diet list for his careful perusal. The list that I have always used is as follows:

DIET LIST.

DO NOT USE: liquors, wine, beer, ale, carbonated waters, or strong tea or coffee, rich cake or candy, new hot bread or pastry, nuts or fried meats, pork, sausage, pickles, shell fish, cheese, cabbage, mushrooms, asparagus, or strawberries.

USE A SIMPLE DIET: such as: fresh meats, eggs, milk, bread and butter, oatmeal, fresh and dried fruits, vegetables and plain food.

Every case of gonorrhea runs a milder course when no alcoholic stimulants are taken. Nevertheless, it is difficult to get patients to abstain from all alcoholic beverages. In the case of elderly men, who have been hard drinkers for many years, sudden abstinance from all alcohol may have a very depressing mental and physical effect. When wine is taken at all, a little red wine can be allowed at meals; but wine always affects the disease unfavorably, even in small quantities.

Too much stress cannot possibly be laid upon the importance of keeping the bowels open. My printed directions to patients are: "Constipation is that condition of the intestines, especially the larger, in which their contents are too firm and are expelled only by strong effort, often painful; the cause being deficient secretion or inaction of the bowels themselves. The result is a diminution in the number and amount of fecal evacuations, headache, flushing of the face, coated tongue, loss of appetite, bad breath, heartburn, sour stomach, a blotched complexion, stomachache, flatulence, a sense of distention, weight in the abdomen, diarrhea from irritation of the lower bowel, mental irritability or depression, displacement of organs in the vicinity of the distended bowel, hemorrhoids, cold feet, swelling of feet and ankles, varicose veins, sciatica and other neuralgias from pressure, and a general poisoning of the system owing to the absorption by the blood of the poisonous matter which should have been excreted. From what has been said the patient can understand that it is a matter of the utmost importance for him to see that his bowels move freely and easily, during the course of his gonorrhea, in order to assist nature to that extent. The patient must therefore acquire the habit of visiting the water-closet once daily and always at the same hour, preferably after breakfast. By degrees the habit will become established, even though at first it seems hopeless. Take time enough, for really this is the most important event of the day. While the habit is being established it may be necessary to use a syringe daily. With a Davidson syringe throw up one or two tumblerfuls of cool water and retain it as long as possible before ejecting it. By thus washing the bowel out at a fixed hour daily the permanent habit is gradually established, and then the use of the syringe may be discontinued. Should the water alone not be sufficient, one or two tablespoonfuls of glycerine may be added to each glass of water. The worst treatment of constipation possible is by strong cathartic medicines, as one soon becomes entirely dependent upon them."

Careless attention to the fundamental details of rest, cleanliness and the daily easy movement of the bowels, will vitiate the results of the most careful local treatment ever given. The physician who would treat gonorrhea successfully must see to it that his hygienic, and dietetic prescriptions, are carefully carried out.

333 Second Avenue.

